# Use of the prostate‐specific antigen (PSA) test in the United States for men age ≥65, 1999–2015: Implications for practice interventions

**DOI:** 10.1002/cnr2.1352

**Published:** 2021-05-01

**Authors:** Shahram Shahangian, Lin Fan, Krishna P. Sharma, David A. Siegel

**Affiliations:** ^1^ Division of Laboratory Systems CDC Atlanta Georgia USA; ^2^ Division of Cancer Prevention and Control CDC Atlanta Georgia USA

**Keywords:** mass screening, prostate‐specific antigen, prostatic neoplasm, public health practice

## Abstract

**Background:**

Various professional organizations have issued recommendations on use of the PSA test to screen for prostate cancer in different age groups.

**Aims:**

Using Medicare claims databases, we aimed to determine rates of PSA testing in the context of screening recommendations during 1999–2015 for US men age ≥65, stratified by age group and census regions, after excluding claims relating to all prostate‐related conditions.

**Methods and Results:**

Medicare claims databases encompassed 9.71–11.12 million men for the years under study. PSA testing rate was the proportion of men with ≥1 test(s) per 12 months of continuous enrollment. Men diagnosed with any prostate‐related condition were excluded. Annual percent change (APC) in PSA test use was estimated using joinpoint regression analysis. In 1999–2015, annual testing rate was 10.1%–23.1%, age ≥85; 16.6%–31.0%, age 80–84; 23.8%–35.8%, age 75–79; 28.3%–36.9%, age 70–74; and 26.4%–33.6%, age 65–69. From 1999 to 2015, PSA testing rate decreased 40.7%, 29.9%, 13.9%, and 2.9%, respectively, for men age ≥85, 80–84, 75–79, and 70–74. For men age 65–69, test use increased by 0.3%. Significant APC trends were: APC_1999–2002_ = +8.1%, *P* = .029 and APC_2008–2015_ = −9.0%, *P* < .001 for men age ≥85; APC_2008–2015_ = −7.1%, *P* = .001 for men age 80–84; APC_2001–2015_ = −2.5%, *P* < .001 for men age 75–79; APC_2008–2015_ = −3.3%, *P* = .007 for men age 70–74; and APC_2010–2015_ = −5.2%, *P* = .014 for men age 65–69.

**Coclusion:**

Although decreased from 1999 to 2015, PSA testing rates remained high for men age ≥70. Further research could help understand why PSA testing continues inconsistent with recommendations.

## 1 INTRODUCTION

Screening recommendations for prostate cancer using the test for prostate‐specific antigen (PSA) have evolved over the past two decades. Although there are a few national estimates of PSA testing rates over recent decades relating to men age ≥65, none have involved an assessment of testing rates and trends over a period of almost two decades, from 1999 to 2015. In 1996, the US Preventive Services Task Force (USPSTF) recommended against routine screening for prostate cancer by using the PSA test.^1^ In 2002, the USPSTF concluded that there was insufficient evidence to make any recommendation to screen for prostate cancer using the PSA test.^2^ In 2008, the USPSTF recommended against screening of men age ≥75 years, with no recommendation for younger men.^3^ In 2012, the USPSTF recommended against PSA screening of men for prostate cancer regardless of age.^4^ Subsequent to the 2012 recommendation, the USPSTF released a draft recommendation in April 2017 noting that men age ≥70 should not be screened, and that the potential benefits and harms of PSA‐based screening were closely balanced in men age 55–69, and that the decision whether to be screened should be an individual one.^5^ The USPSTF issued its most recent recommendations in May 2018,^6^ that was identical to the 2017 draft recommendations.^5^ The American Urological Association (AUA)^7^ and American College of Physicians (ACP)^8^ in 2013 both recommended against routine screening of men age ≥70. Like the USPSTF,^6^ the AUA recommended that the decision for men age 50–69 to submit to screening be an individual one^7^ and the ACP recommended individualized screening in this age group be limited to those with at least 10 years of life expectancy.^8^ In 2016, the American Cancer Society (ACS) stipulated a starting age of 50 for individualized PSA‐based screening of men with at least 10 years of life expectancy.^9^ In 2001–2002, the AUA, ACP, and ACS all included informed and shared decision‐making, with the AUA and ACP recommending a starting age of 50.^10^

Using Medicare claims databases in the context of screening recommendations for prostate cancer, our objective was to determine the rates and trends for annual PSA testing in US men age ≥65 in 1999–2015, stratified by age group, after excluding claims relating to all prostate‐related conditions. Annual testing rates and trends were examined for different age groups overall and after stratifying by the four US Census regions. These analyses were undertaken to assess PSA screening uptake in older men of different age groups and particularly those age ≥70 for whom routine PSA screening is not recommended. Information gained from this study may be used to inform current practice interventions.

## 2 METHODS

### 2.1 Population

We used 100% Career files for Medicare claims data for men age ≥65 from January 1, 1999 through December 31, 2015, encompassing 9.71–11.12 million men with continuous enrollment for each calendar year. We excluded all men with claims containing one or more diagnoses or non‐laboratory procedures relating to various prostate‐related diseases or conditions, including those diagnostic codes related to elevated PSA or history of prostate cancer (see Appendix). Although men with nonmalignant prostate‐related diseases or conditions may indeed undergo PSA testing for screening purposes, it is likely that some may have undergone PSA testing in the context of differential diagnosis in order to rule out prostate cancer. We therefore opted to conservatively exclude all populations with encounter claims for PSA testing that may have been performed for differential diagnosis, monitoring, or prognostic purposes. We included only claims with a Current Procedural Terminology (CPT, American Medical Association) Code 84152 for complexed PSA and Code 84153 for total PSA, and Healthcare Common Procedure Coding System (HCPCS, Centers for Medicare and Medicaid Services) code G0103 for total PSA testing. We did not use CPT code 84154 for free PSA because this test, unlike total or complexed PSA, is not initially used to screen for prostate cancer. If PSA test result is in the borderline range, usually considered 4.0–10.0 μg/L, free/total PSA ratio may be used to decide if a prostate biopsy is warranted.

### 2.2 Annual PSA testing rates

Annual rates for PSA testing in each calendar year were the proportion of the study population with ≥1 PSA test(s) per 12 months continuous enrollment after excluding those with any prostate‐related condition.

### 2.3 Annual percent change and PSA testing trends

Annual percent change (APC) was used to estimate the change in PSA test use. APC was estimated using joinpoint regression analysis, fitting trend data to identify the log‐linear model with the fewest number of inflection points.^11^ Therefore, the software used fits trend data using a regression model that minimizes the number of inflection points, and therefore the number of APC line segments. APC was the log‐linear slope of each trend line, and *P* values related to statistical significance of each APC estimate being different from zero.

### 2.4 Stratification scheme

We stratified the study population into different age groups: 65–69, 70–74, 75–79, 80–84, and ≥85. For each age group, we further stratified data into the four US Census regions (https://www.census.gov/prod/1/gen/95statab/preface.pdf): Northeast (CT, MA, ME, NH, NJ, NY, PA, RI, and VT), Midwest (IA, IL, IN, KS, MI, MN, MO, OH, NE, ND, SD, and WI), South (AL, AR, DC, DE, FL, GA, KY, LA, MD, MS, NC, OK, SC, TN, TX, VA, and WV), and West (AK, AZ, CA, CO, HI, ID, MT, NM, NV, OR, UT, WA, and WY). This stratification scheme was used to better understand the extent of overall and regional variation of PSA test use by age.

## 3 RESULTS

### 3.1 Included populations

The final study sample included 6.46–7.79 million men in each year from 1999 through 2015 after excluding men having encounter claims associated with prostate‐related diseases or conditions (see Methods). As the result of this exclusion, 66.5%–70.1% of men remained for analysis of PSA testing rates and trends.

### 3.2 Annual PSA testing rates and percent changes

Annual rate of PSA testing ranged from 10.1%–23.1% in men age ≥85 to 28.3%–36.9% in men age 70–74. PSA testing rate decreased by 2.9%, 13.9%, 29.9%, and 40.7%, respectively, for men age 70–74, 75–79, 80–84, and ≥80 from 1999 to 2015. However, testing rate increased by 0.3% for men age 65–69 from 1999 to 2015. Table 1 shows the range of annual PSA testing rate and annual percent change (APC) for men in each of these five groups.

**TABLE 1:** Annual PSA testing rate and annual percent change (APC)

Age	Testing rate	APC	Years	*P*
65–69	26.4%–33.6%	+1.0%	1999–2010	.10
		−5.2%	2010–2015	.014
70–74	28.3%–36.9%	+6.3%	1999–2002	.063
		−6.6%	2002–2005	.27
		+5.5%	2005–2008	.40
		−3.2%	2008–2015	.007
75–79	23.8%–35.8%	+9.6%	1999–2001	.24
		−2.4%	2001–2015	<.001
80–84	16.6%–31.0%	+7.0%	1999–2002	.053
		−8.9%	2002–2005	.16
		+3.6%	2005–2008	.59
		−6.8%	2008–2015	<.001
≥85	10.1%–23.1%	+8.1%	1999–2002	.029
		−9.8%	2002–2005	.14
		+1.0%	2005–2008	.87
		−9.0%	2008–2015	<.001

### 3.3 Annual PSA testing trends for men age ≥65

**FIGURE 1:**
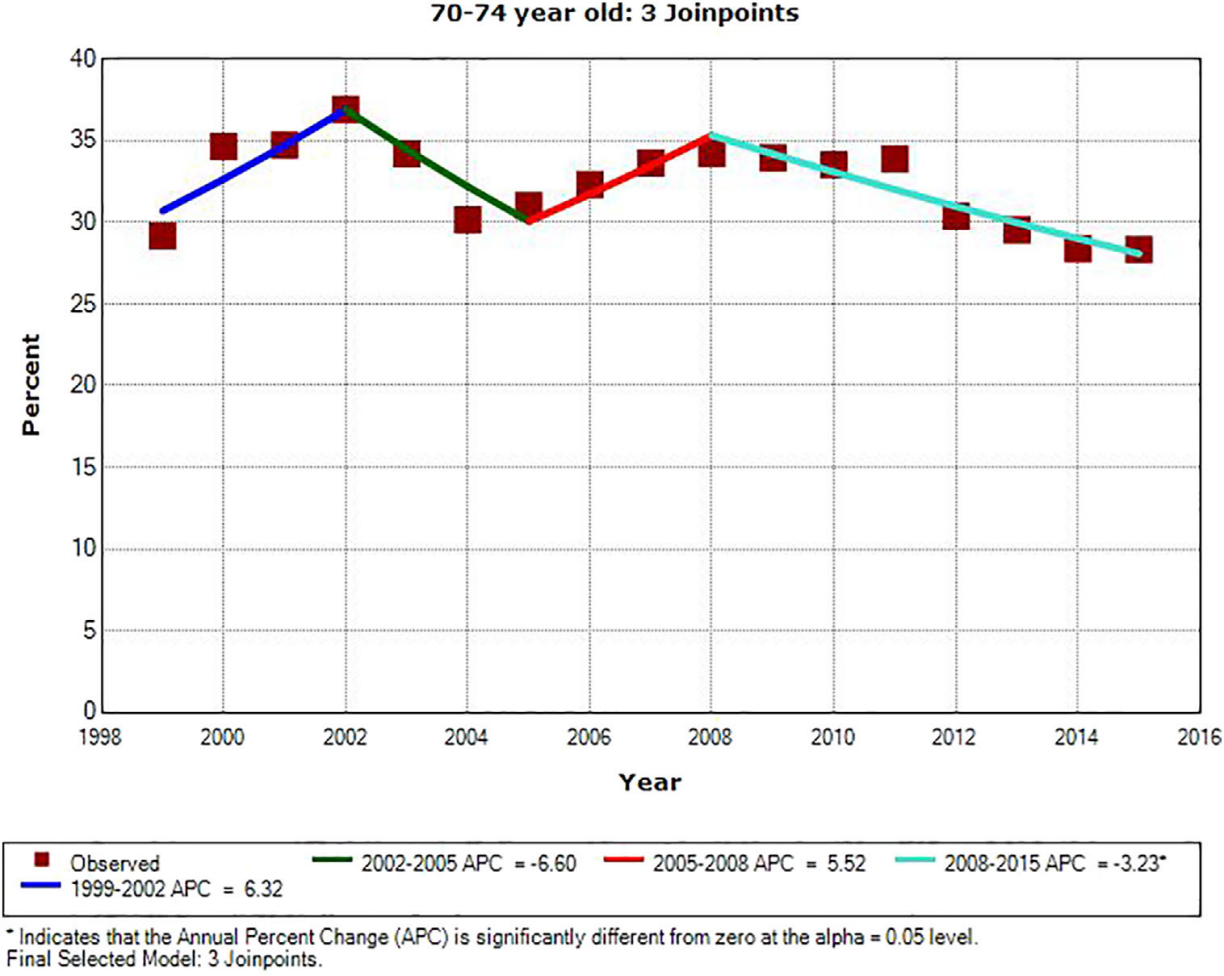
Annual PSA testing rates and trend in men age 70–74. *APC is significantly different from zero (*P* < .05)

**FIGURE 2:**
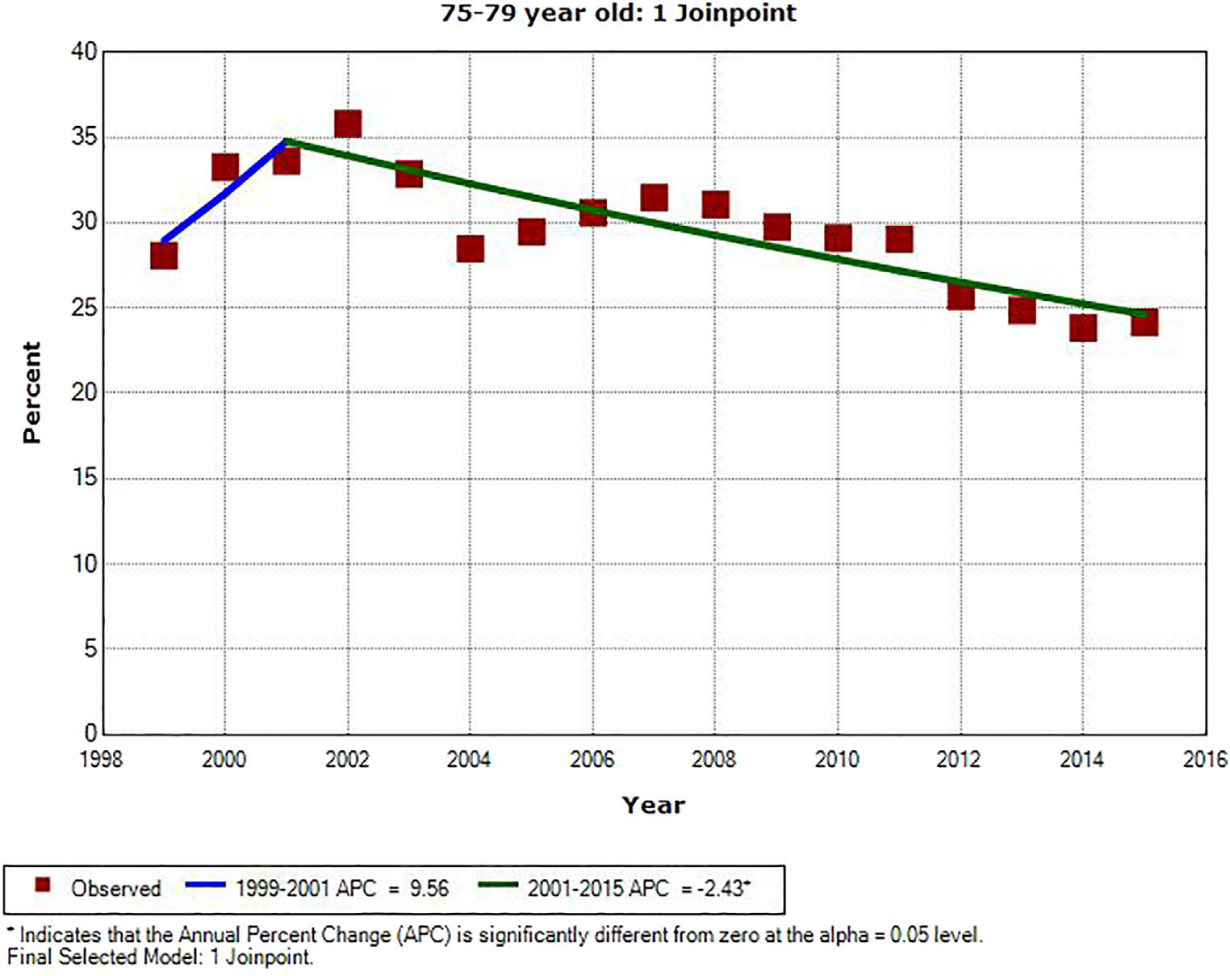
Annual PSA testing rates and trend in men age 75–79. *APC is significantly different from zero (*P* < .05)

**FIGURE 3:**
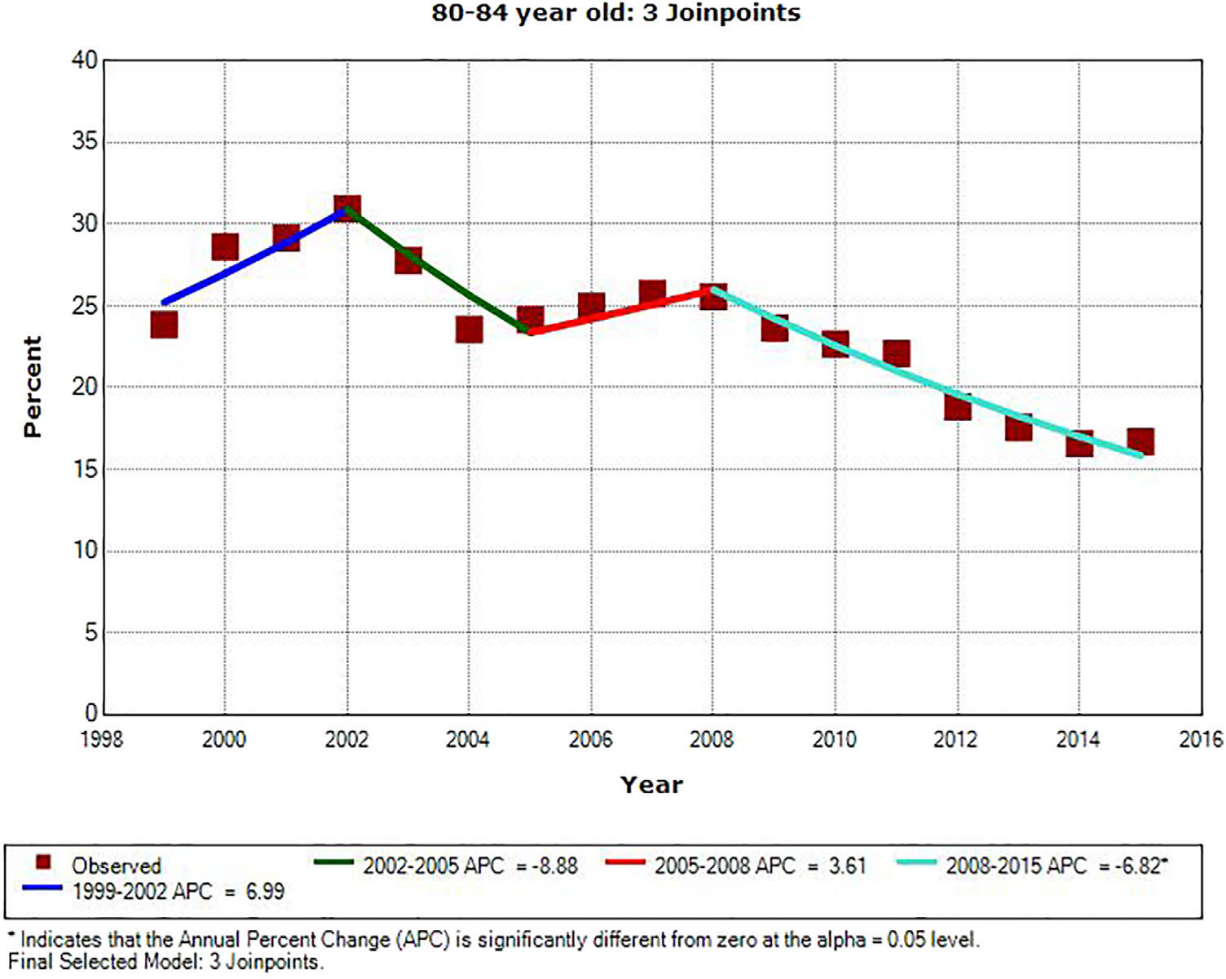
Annual PSA testing rates and trend in men age 80–84. *APC is significantly different from zero (*P* < .05)

**FIGURE 4:**
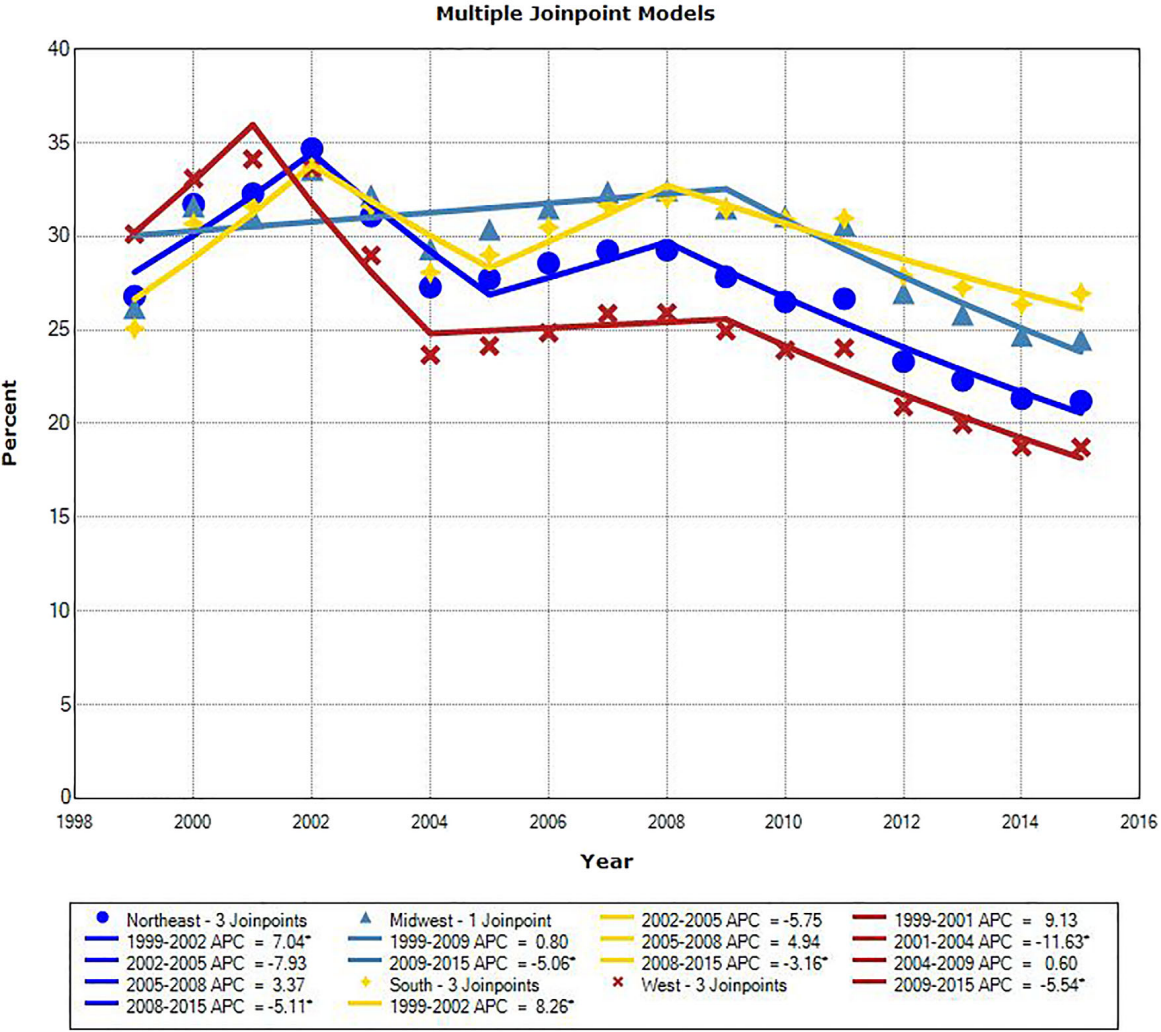
Annual PSA testing rates and trend in men age ≥65 stratified by census region. *APC is significantly different from zero (*P* < .05)

There were significant downward trends in PSA testing rate for all age groups during the most recent years of the study period. For men ages 65–69 and 75–79, the APC was −5.2% from 2010 to 2015 (*P* = .014) and −2.4% from 2001 to 2015 (*P* < .001), respectively. For the other three age groups, the last APC line segment was from 2008 to 2015. The APC value was −3.2% in men age 70–74 (*P* = .007), and it was −6.8% and −9.0% in men ages 80–84 and ≥85, respectively (*P* < .001). There were three inflection points for all but two age groups, ages 65–69 and 75–79, for which we identified only one inflection point. The slopes of APC line segments alternated from positive to negative, starting positive and ending negative. The only other significant APC was for men age ≥85 from 1999 to 2002; the APC value was +8.1% (*P* = .029). The three inflection points for men ages 70–74, 80–84, and ≥85 all occurred in the same years: 2002, 2005, and 2008. The one inflection point for men ages 65–69 and 75–79 were in 2010 and 2001, respectively. The trends of annual PSA testing rates for men age ≥70, who should not be routinely screened for prostate cancer, are shown in three age groups of 70–74, 75–79, and 80–84 in Figures 1, 2, and 3, respectively. Regional PSA testing trends are shown in Figure 4 for all male Medicare enrollees age ≥65.

Annual PSA testing rates and their trends in men age ≥65 are shown in Figure 4, after stratifying them by US Census region. In later years, the highest annual testing rates were seen in the South, while the lowest rates were in the West. Except for one region with one inflection point in 2009 (Midwest), the other three regions exhibited three inflection points. These occurred in 2001–2002, 2004–2005, and 2008–2009.

## 4 DISCUSSION

This study of Medicare claims data going back to 1999 shows that the PSA test has been used among men in all age groups over 65, including those 70 and older. Although the testing rate for this age group has been decreasing in the most recent years, the testing rate in 2015 still ranged from 10% for men age ≥85 to 28% for those age 70–74. Other studies have reported annual PSA testing rates for US men age ≥65 using electronic records (2002–2014),^12^, ^13^, ^14^, ^15^, ^16^, ^17^, ^18^, ^19^ claims data (2006–2013),^20^, ^21^, ^22^ and self‐reported data obtained from the CDC's National Health Interview Surveys (NHIS) in 2000–2015^23^, ^24^, ^25^, ^26^, ^27^, ^28^, ^29^, ^30^, ^31^, ^32^, ^33^ and Behavioral Risk Factor Surveillance Systems (BRFSS) in 2006–2012.^34^, ^35^, ^36^ All of these studies show that at least 20% of men age ≥70 have undergone PSA testing annually since 2000. Annual PSA testing rates and trends reported in these studies are consistent with our findings for 1999–2015. Our results, consistent with those previously reported, show an increase in PSA testing rate from 1999 to 2002^31^; and after decreasing from 2002 to 2005, PSA testing rates exhibit an increase again from 2005 to 2008.^29^, ^31^ All studies, including ours, consistently show a significant decrease in PSA testing rate from 2008 to 2013–2015.^12^, ^13^, ^15^, ^21^, ^22^, ^23^, ^24^, ^27^, ^29^, ^30^, ^31^ Our study is unique, however, in that it is the only one that is based on the largest claims databases relating to men age ≥65 over the longest period of observation, from 1999 through 2015, while using the most comprehensive diagnostic list of prostate‐related conditions to exclude encounters that may be for PSA testing for purposes other than screening for prostate cancer. Most studies of PSA testing in these age groups are based on self‐reports, and the few claims‐based studies are limited to only a few years, and do not cover the entire Medicare population over such an extended period of time between 1999 to 2015 . Unique to this study is the exclusion of enrollees and PSA testing events associated with any prostate‐related diagnoses and procedures that may have been for purposes other than screening. Also unique to this study is the consistent regional differences in PSA testing rates between 1999 and 2015.

Most studies reporting on PSA testing rates in men age ≥70 have been based on self‐reported NHIS and BRFSS surveys.^23^, ^24^, ^25^, ^26^, ^27^, ^28^, ^29^, ^30^, ^31^, ^32^, ^33^, ^34^, ^35^, ^36^ Test utilization rates obtained from such surveys are, however, subject to recall, nonresponse and framing biases. Such rates obtained from claims data, on the other hand, are not subject to these biases. Nevertheless, testing events obtained from administrative databases cannot be equated to actual testing events because adjudicated and paid claims may not include some tests that were performed but not reimbursed. Despite this inherent limitation in using administrative databases to determine actual test utilization rate, claims data may provide more objective information than survey data. This is because test utilization rates obtained from claims databases are not subject to the aforementioned biases pertaining to NHIS and BRFSS surveys. To the best of our knowledge, this study is the only one based on nationwide claims data for men age ≥65 that encompass a 17‐year period from 1999 to 2015. Goodwin et al used Texas Medicare claims data for men age ≥75 in 2007 and 2010.^20^ Howard et al used national Medicare claims data for the same age group in 2006–2010, showing a 7.9% decrease in PSA testing from 2008 to 2010.^21^ Kim et al used claims data from Optum Lab Data Warehouse in 2008–2013, and demonstrated that PSA decreased significantly during this period.^22^ PSA testing rates obtained from electronic data in 2007–2014 also show decreasing PSA testing rates in men age ≥70.^12^, ^13^, ^15^ However, none of these studies cover the period from 1999 to 2015 for the entire Medicare populations of men age ≥65, while comprehensively excluding all encounters with diagnoses of any prostate‐related conditions.

Like Walter et al,^18^ we excluded, but more comprehensively, enrollees with encounter claims for PSA tests that may have been used for purposes other than screening, such as monitoring of those with a history of prostate cancer or other prostate‐related health conditions. However, despite the exclusion criteria applied, the current study may include some patients who received PSA testing for reasons other than screening. Because we do not know how commonly PSA tests are ordered for purposes other than screening in the included population, unlike other investigators,^18^, ^24^, ^25^, ^26^ we have equated captured claim events to PSA testing, and not to PSA‐based screening. After exclusion of claims associated with prostate‐related conditions, still 67%–70% of Medicare enrollees remained. Because PSA‐based screening is not expected to be performed more than once or at most twice per year, we used another claims database, MarketScan, and determined that the vast majority (97%–99%) of the included population had ≤2 PSA tests per year consistent with PSA testing being used for screening (unpublished observation).

There were consistent geographic differences in annual PSA testing rates in the four US Census regions, with the West exhibiting the lowest PSA testing rates (2003–2015) and the South showing the highest (2011–2015) in recent years. These findings are consistent with PSA testing rates we have observed for men age 55–64 from 2011 to 2017 using MarketScan claims data, showing that the highest PSA testing rates were in the South and the lowest rates in the West.^37^

In 2013, it was estimated that 1.4 million men age ≥65 with a >52% risk of 9‐year mortality were screened with the PSA test.^24^ In 2005, 31% of men with low‐life expectancy (>48% probability of death within 10 years) had a PSA‐based screening.^25^ In 2010, 34% of men age ≥75 with a >75% predicted 9‐year mortality were screened for prostate cancer with the PSA test.^26^ Approximately, 55% of screened men age ≥75 who had ≥53% predicted 9‐year mortality recalled discussing advantages of screening, whereas only 25% recalled discussing disadvantages. Given these data and the cost implication of PSA‐based screening,^38^, ^39^ effective strategies could be developed to decrease PSA‐based screening inconsistent with current recommendations. Trends data show, however, that the PSA testing rates are decreasing with advancing age for men age ≥70. Although individualized PSA testing with shared decision making is primarily advocated for men age 50/55 to 69, some providers may extend testing to men older than 69. Many clinicians may choose to personalize screening recommendations by conducting PSA testing in men age ≥70 with no significant comorbidities and a life expectancy of ≥10 years. Our analyses show how prevalent PSA screening is in different age groups of men age ≥70. Although the USPSTF does not include in their screening recommendations known risk factors other than age, such as race and family history, these may impact the decision to test for PSA. Determination of testing rate in higher‐risk groups, such as African American men, was outside the scope of this study. This may be an avenue for future investigation. However, a limitation of using Medicare claims data is that African American men constitute a small proportion of men age ≥70. PSA testing rates in the four census regions showed consistent differences among them over the years that can be influenced by various populations characteristics including risk factors, as well as regional differences in screening practices.

In summary, our analyses show that annual rate of PSA testing, likely done to screen for prostate cancer, has significantly decreased for all age groups from 2008 to 2015. PSA testing rate decreased with advancing age in men age ≥70. However, a substantial proportion of men age ≥70 still underwent PSA testing: from approximately 10% in men age ≥85 in 2015 to 28% in men age 70–74. These findings may have implications for better use of the PSA test in clinical practice. To do so, future research could help to understand why PSA tests continue to be ordered for men age ≥70, many with <10 years of life expectancy. Ordering of a laboratory test is a pre‐analytic component of the total testing process, and appropriate laboratory test utilization is a vital component of laboratory quality and diagnostic excellence. Continued monitoring could show if the decreasing trend in PSA testing continues in alignment with current evidence‐based recommendations against routine PSA‐based screening of men age ≥70.

## CONFLICT OF INTEREST

The authors have stated explicitly that there are no conflicts of interest in connection with this article.

## AUTHOR CONTRIBUTIONS

Shahram Shahangian: concept and design of the study, data analysis, interpretation of data, drafting of the initial manuscript, review and revision of the manuscript, approval of the final manuscript as submitted, and accountability for all aspects of the work. Lin Fan and Krishna P. Sharma: concept of the study, data curation, interpretation of data, review and revision of the manuscript, approval of the final manuscript as submitted, and accountability for all aspects of the work. David A. Siegel: concept of the study, interpretation of data, review and revision of the manuscript, approval of the final manuscript as submitted, and accountability for all aspects of the work.

## ETHICS STATEMENT

Institutional clearance and approval was obtained prior to submission.

## Data Availability

The data that support the findings of this study are available from the corresponding author upon reasonable request.

## Appendix

**Table:**

Code	Source	Description
60.21	ICD‐9	Transurethral ultrasound‐guided, laser induced prostatectomy
60.29	ICD‐9	Other transurethral prostatectomy
60.3	ICD‐9	Suprapubic/transvesical prostatectomy
60.4	ICD‐9	Retropubic prostatectomy
60.61	ICD‐9	Local excision of lesion of prostate
60.62	ICD‐9	Perineal prostatectomy; cryoablation of prostate; cryoprostatectomy and surgery of prostate
60.69	ICD‐9	Other operations on prostate and seminal vesicles
185.0	ICD‐9	Malignant neoplasm of prostate
222.2	ICD‐9	Benign neoplasm of prostate
233.4	ICD‐9	Carcinoma *in situ* of prostate
236.5	ICD‐9	Neoplasm of uncertain behavior of prostate
600.00	ICD‐9	Benign hypertrophy of prostate without urinary obstruction and other LUTS
600.01	ICD‐9	Benign hypertrophy of prostate with urinary obstruction and other LUTS
600.10	ICD‐9	Nodular prostate without urinary obstruction
600.11	ICD‐9	Nodular prostate with urinary obstruction
600.20	ICD‐9	Benign localized hyperplasia of prostate without urinary obstruction and other LUTS
600.21	ICD‐9	Benign localized hyperplasia of prostate with urinary obstruction and other LUTS
600.90	ICD‐9	Hyperplasia of prostate, unspecified, without urinary obstruction and other LUTS
600.91	ICD‐9	Hyperplasia of prostate, unspecified, with urinary obstruction and other LUTS
601.0	ICD‐9	Acute prostatitis
601.1	ICD‐9	Chronic prostatitis
601.2	ICD‐9	Abscess of prostate
601.3	ICD‐9	Prostatocystitis
601.4	ICD‐9	Prostatitis in diseases classified elsewhere
601.8	ICD‐9	Other specified inflammatory diseases of prostate
601.9	ICD‐9	Prostatitis, unspecified
602.0	ICD‐9	Calculus of prostate
602.1	ICD‐9	Congestion or hemorrhage of prostate
602.3	ICD‐9	Dysplasia of prostate
602.8	ICD‐9	Other specified disorders of prostate
602.9	ICD‐9	Unspecified disorder of prostate
790.93	ICD‐9	Elevated PSA
55801	ICD‐9	Perineal subtotal prostatectomy
55810	CPT	Perineal radical prostatectomy
55812	CPT	Perineal radical prostatectomy with lymph node biopsies
55815	CPT	Perineal radical prostatectomy with bilateral pelvic lymphadenectomy
55821	CPT	Suprapubic, subtotal prostatectomy
55831	CPT	Retropubic, subtotal prostatectomy
55840	CPT	Retropubic radical prostatectomy with or without nerve sparing
55842	CPT	Retropubic radical prostatectomy with or without nerve sparing with lymph node biopsies
55845	CPT	Retropubic radical prostatectomy with(out) nerve sparing/lymph node biopsies with lymphadenectomy
55866	CPT	Laparoscopy, retropubic radical prostatectomy including nerve sparing/robotic assistance

Abbreviations: CPT, current procedural terminology; ICD, International Classification of Disease; LUTS, lower urinary tract symptom.
